# Fluctuations and extreme events in the public attention on Italian legislative elections

**DOI:** 10.1038/s41598-024-69354-y

**Published:** 2024-10-01

**Authors:** Andrea Auconi, Lorenzo Federico, Gianni Riotta, Guido Caldarelli

**Affiliations:** 1 Department of Molecular Sciences and Nanosystems, Ca’ Foscari University of Venice, 30172 Venice, Italy; 2 LUISS Data Lab, Viale Pola 12, 00198 Rome, Italy; 3 Department of Political Sciences, LUISS University, Viale Romania 32, 00197 Rome, Italy; 4 European Centre for Living Technology, 30124 Venice, Italy; 5 Institute for Complex Systems, Consiglio Nazionale delle Ricerche, UoS Sapienza, 00185 Rome, Italy; 6 London Institute for Mathematical Sciences, Royal Institution, London, W1K2XF UK

**Keywords:** Statistical physics, Applied mathematics

## Abstract

The share of social media attention to political candidates was shown to be a good predictor of election outcomes in several studies. This attention to individual candidates fluctuates due to incoming daily news and sometimes reflects long-term trends. By analyzing Twitter data in the 2013 and 2022 election campaign we observe that, on short timescales, the dynamics can be effectively characterized by a mean-reverting diffusion process on a logarithmic scale. This implies that the response to news and the exchange of opinions on Twitter lead to attention fluctuations spanning orders of magnitudes. However, these fluctuations remain centered around certain average levels of popularity, which change slowly in contrast to the rapid daily and hourly variations driven by Twitter trends and news. In particular, on our 2013 data we are able to estimate the dominant timescale of fluctuations at around three hours. Finally, by considering the extreme data points in the tail of the attention variation distribution, we could identify critical events in the electoral campaign period and extract useful information from the flow of data.

## Introduction

The recent technological revolution made it possible to store unprecedented information regarding society. Data from all kinds of media, including e-mails, phone calls, online purchases, travel info and more, are stored, forming what has been called the data deluge^1^. This availability of data has offered more opportunities for advanced mathematical techniques to be applied in the modeling of social and political phenomena, towards a quantitative description of human behaviour and its multi-layer interactions within society^2–4^. The classical definition of complexity as “more is different”^5^ manifests here in the fact that social interactions, which typically involve just two or only a few individuals, can propagate their effect through a network to generate collective behaviour in a way that is reminiscent of phase transitions in statistical physics^6,7^.

Methods of statistical physics and network theory^8,9^ have already found application in social sciences for the study of information spreading and fake news^10–12^, and in particular to characterise the relation between news diffusion properties and the underlying network structure. The latter is usually inferred from interactions on platforms like Twitter and Facebook. On Twitter, the clustering around influential people is particularly strong^13^, as well as the emergence of closed “echo chambers” of people mutually reinforcing their opinions^14,15^, so that a coarse-grained description in terms of communities and narratives was possible. In particular, studies based on Twitter data elucidated contagion mechanisms in the Arab Spring demonstrations^16^, Occupy Wall Street^17^, and the Spanish “Indignados”^18^. Also, many tried to extract information from social networks to get some anticipation on the outcome of online electoral campaigns in various countries including USA^19–25^, Australia^26,27^, Norway^28^, Spain^29^, Italy^30,31^, France^32^, and UK^33^.

In this paper, we study the fluctuation properties of the attention towards political parties on Twitter in the weeks preceding the Italian political elections of 2013 and 2022. In particular our question is: how can we improve our models of stochastic varying attention online, so that we can better connect this to election outcomes and possibly other phenomena? We find that, on short timescales of minutes up to a few days, the dynamics is well described by a mean-reverting diffusion process on a logarithmic scale, meaning that the reaction to news and exchange of opinions on Twitter produces fluctuations of attention varying over orders of magnitudes but still anchored around some set of average levels of popularity which change only slowly compared to the daily and hourly variations due to news and trends on Twitter. We find that this minimal description employing just two parameters, one for the timescale of mean-reversion and another for the amplitude of fluctuations, is sufficient to interpolate the average squared logarithmic displacement of the share of Twitter attention, which is a basic measure of uncertainty on the future level of attention. By calibrating this model to our time series data we find that the dominant timescale of fluctuations can be estimated at around three hours.

Regarding the distribution of these squared log displacements, however, we observe some larger tails than the Gaussian model would typically show. Indeed, like in many other social phenomena, we also find some extreme events, here as short periods of collective attention^34^. These are relatively rare and happen in a small absolute number in our data. Therefore, further modeling in terms of a jump-diffusion process and a fat-tailed distribution is not statistically feasible. Still, we investigate these individual jumps in attention and find that they correspond to significant unexpected events in the political campaign. Our analysis of fluctuations on short timescales of minutes up to a few days complements the studies of trends and inertia in the attention dynamics over longer timescales^31,35–41^.

## Methods

### The datasets

The first and main dataset used here consists of around 3.5 M tweets recorded from 1 January 2013 to 22 February 2013 before the general italian political elections which took place on the 24 and 25 February 2013. This dataset was created and already used for a different analysis in^31^. Tweets were selected using the Twitter free API in *stream* mode with italian language as only filter, and then classified based on if they mention political leaders’ family names or their political party with or without hashtag. The free API provides only a subset of the whole tweets population, and this determines an increased estimation noise especially relevant for small filtering timescales as discussed below. Similarly the second dataset for the general italian political elections of 25 September 2022 consists of around 2.5 M Tweets recorded from 6 September 2022 to 23 September 2022 selected according to a list of hashtags provided in^42^, and then classified based on if they mention the leaders and the six main political parties, such keywords provided in the Supplementary file “Keywords”.

### The share of Twitter attention

Our data consists of a time series of tweets regarding politicians in Italy during the weeks preceding the political elections of 2013 and 2022. For a given political candidate *i* the time series is a list of events’ times $\left[ t_1, t_2, \dots , t_{n_i} \right]$ corresponding to tweets mentioning that particular candidate or its political party. We express this equivalently with a measurement process *m*(*t*) making unit jumps for each event, namely $dm(t)=1$ if *t* is in the list of events’ times and $dm(t)=0$ otherwise. The differentials $dm(t)=1$ can be interpreted as the limit of a time discretization of vanishing size. We wish to estimate an instantaneous twitting rate *y*(*t*) from the measurement process *m*(*t*), by using known results in the field of stochastic dynamics. Indeed, this problem is generally formulated in the language of stochastic filtering^43,44^, where a likelihood function is dynamically updated with respect to a model for the stochastic dynamics of the unobserved process *y*(*t*) and its measurement probability *p*(*dm*|*y*). However, we wish not to make strong modeling assumptions at this stage, therefore we considered a linear filter of the kind

1 $$\begin{aligned} y(t) = \beta \int ^t_{-\infty } dm(t')\, e^{-\beta (t-t')}, \end{aligned}$$

with a timescale $\beta ^{-1}$ which should be large enough to contain a sufficiently large number of events (for a precise rate estimation), but also sufficiently small compared to the natural time of tweet rate variations. As the latter is not yet determined, we practically fix $\beta ^{-1} = 20$ minutes and later vary it to check that results do not significantly depend on it. Similarly, the results should be invariant if taking a time binning approach with size $\beta ^{-1}$ instead of the corresponding linear filter. Let us denote the expectation value of a quantity as $\mathbb {E}[\dots ]$. We can express Eq. (1) equivalently in differential form as $dy = \beta (dm - y\, dt)$, and we see that if the twitting rate is constant, i.e. $\mathbb {E} [dm] = r \, dt$, then at steady state ($\mathbb {E}[dy] = 0$) we have $\mathbb {E} [y] = r$ as expected. The fluctuations around this average level due to the discreteness of events is calculated as $\textrm{Var} [y] = r \beta /2$.

We are here interested in the share of Twitter attention on the various political candidates, as this may indicate voting intentions in the population. Therefore we normalize each $y_i$ by its total among the candidates by defining $x_i = y_i/ \left( \sum _i y_i \right)$. This definition of $x_i$ as a share of Twitter attention also works as a de-trending mechanism. The twitting rate has a prominent daylight dependence since the activity is much reduced at night. While some noise due to the lower rates in the night affects *x*, we nevertheless check the stability of our results by removing some of the night hours from the *y* integral. Finally, let us note that considering the twitting rate in relative terms has the consequence of showing apparent drops due to spikes in other processes.

### Geometric Ornstein–Uhlenbeck process

Consider the following stochastic differential equation in the Itô interpretation^45^,

2 $$\begin{aligned} dx = \sigma x dW - \alpha x \ln \left( \frac{x}{\mu } \right) dt, \end{aligned}$$

where *dW* denote Brownian motion increments satisfying $\mathbb {E} \left[ dW(t) \right] = 0$, $\,\mathbb {E}\left[ dW(t) dW(t') \right] = \delta _{t t'} dt$, and the Dirac delta is here $\int _0^T \delta _{t t'} =\mathbb {I}_{t\in [0,T]}$ in terms of the indicator function $\mathbb {I}_{t\in [0,T]}$. Using Itô’s Lemma^45^ we find

3 $$\begin{aligned} d\ln x = \sigma dW - \alpha \ln \left( \frac{x}{\mu } \right) dt -\frac{\sigma ^2}{2} dt , \end{aligned}$$

that can be rewritten in terms of $\widetilde{x}\equiv \ln x$ as

4 $$\begin{aligned} d \widetilde{x} = \sigma dW - \alpha \left( \widetilde{x} -\widetilde{\mu } \right) dt, \end{aligned}$$

where $\widetilde{\mu } = \ln \mu -\frac{\sigma ^2}{2\alpha }$. We see from the definition of $\widetilde{x}$ and its dynamics in Eq. (4) that *x* follows an Ornstein–Uhlenbeck process^46^ in logarithmic scale, which we here call Geometric Ornstein–Uhlenbeck (GOU) process. The GOU process combines the logarithmic scale diffusion of the geometric Brownian motion with the linear mean-reverting property of the Ornstein–Uhlenbeck process. These two features are governed by the volatility parameter $\sigma$ and the inverse correlation time $\alpha$. The Ornstein–Uhlenbeck process admits a stationary Gaussian density $p( \widetilde{x} ) = \mathcal {N}(\widetilde{\mu },\frac{\sigma ^2}{2 \alpha })$ with mean $\mathbb {E}[\widetilde{x}] = \widetilde{\mu }$ and variance $\mathbb {E}\left[ \widetilde{x}^2 \right] -\left( \mathbb {E}\widetilde{x} \right) ^2 = \frac{\sigma ^2}{2 \alpha }$. The average level of the *x* process is given by the Gaussian integral $\mathbb {E} [x] = \int d\widetilde{x} \, p( \widetilde{x} ) \, e^{\widetilde{x}} = \mu \,e^{-\frac{\sigma ^2}{4\alpha }}$.

### The squared log-displacement

Let us consider the squared log-displacement variable

5 $$\begin{aligned} s(\tau ) = \left[ \ln \left( \frac{x(\tau )}{x(0)}\right) \right] ^2 , \end{aligned}$$

where for simplicity we drop the explicit dependence on *t* and imagine to sample the process at $t=0$ from the stationary density. From the above definitions we can write $s(\tau )=\left( \widetilde{x}(\tau )-\widetilde{x}(0)\right) ^2$, consider the well-known solution to the Ornstein–Uhlenbeck process,

6 $$\begin{aligned} \widetilde{x}(\tau ) = \left( \widetilde{x}(0) -\widetilde{\mu } \right) e^{-\alpha \tau } + \widetilde{\mu } + \sigma \int _0^{\tau } e^{-\alpha (\tau -t)} dW(t) , \end{aligned}$$

and use it to evaluate Eq. (5). Then noting that $\mathbb {E} \left[ \left( \widetilde{x}(0) -\widetilde{\mu } \right) ^2 \right] = \frac{\sigma ^2}{2\alpha }$ in the stationary density, and computing the expectations as usual from the Brownian motion covariance, we obtain

7 $$\begin{aligned} \mathbb {E} \left[ s(\tau )\right] = \frac{\sigma ^2}{\alpha } (1-e^{-\alpha \tau }) , \end{aligned}$$

which characterizes the scaling of the mean square log-displacement with the time interval $\tau$ and its exponential saturation due to the linear mean-reversion.

### Fitting the data

The quantity *x*(*t*) that we choose to analyze the Twitter share of attention dynamics is the empirical average square log displacement. Accordingly, the model to interpret its dependence on the time interval is the GOU process described above. In particular, we calibrate the two parameters $\sigma$ and $\alpha$ in the theoretical curve of Eq. (7), $\left. {{\mathbb{E}}\left[ {s(\tau _{j} )} \right] \equiv {\mathbb{E}}\left[ {s(\tau _{j} )} \right]} \right|_{{\sigma ,\alpha }}$, from samples of the empirical average square log-displacement $\widetilde{\mathbb {E}} \left[ s(\tau _j)\right]$ for a set of time intervals, $\tau _j = \tau _0 \lfloor 1.2^j\rfloor$, with the shortest $\tau _0 = 30$ min taken large enough so that the impact of the discreteness of events is small, and up to $\tau _j < 30$ hours as to be still much smaller than the total observation period. We also require $\tau _0>\beta ^{-1}$ to reduce the overlapping of the exponential kernels in the tweet rate estimation. The impact of the discreteness can then be taken into account approximately (by neglecting normalization correlations) to first order in the empirical average rate $\widetilde{\mathbb {E}} [y_i]$ and variance $\widetilde{\textrm{Var}}[y_i]$ by subtracting to the empirical curve $\widetilde{\mathbb {E}} \left[ s(\tau _j)\right] \rightarrow \widetilde{\mathbb {E}} \left[ s(\tau _j)\right] -\widetilde{\mathbb {E}} \left[ s\right] ^{(b)}$ a background noise

8 $$\begin{aligned} \widetilde{\mathbb {E}} \left[ s\right] ^{(b)}=\frac{\beta }{\widetilde{\mathbb {E}} [y_i]}\left( 1+\frac{\widetilde{\textrm{Var}}[y_i]}{(\widetilde{\mathbb {E}} [y_i])^2} \right) , \end{aligned}$$

where the Log-Normal distribution for *y* was assumed.

The fit is then performed by minimizing the mean squared error, $\min _{{\sigma ,\alpha }} \sum\nolimits_{j} {\left( {\left. {{\tilde{\mathbb{E}}}\left[ {s(\tau _{j} )} \right] - {\mathbb{E}}\left[ {s(\tau _{j} )} \right]} \right|_{{\sigma ,\alpha }} } \right)} ^{2}$.

Once the parameters $\widetilde{\sigma }_i$ and $\widetilde{\alpha }_i$ are fitted for each political candidate *i*, we study if the discrepancy from the fitted curve has some common structure, which would signal for improper modeling assumptions. This is done by normalizing the empirical expectation $\widetilde{\mathbb {E}} \left[ s(\tau _j)\right] _i$ for each candidate *i* by the fitted $\frac{\widetilde{\sigma }^2_i}{\widetilde{\alpha }_i}$, and then rescaling the time intervals differently for each candidate by the fitted $\widetilde{\alpha }_i$, namely

9 $$\begin{aligned} z_i(\widetilde{\alpha }_i\tau _j) = \widetilde{\mathbb {E}} \left[ s(\tau _j)\right] _i \frac{\widetilde{\alpha }_i}{\widetilde{\sigma }^2_i} , \end{aligned}$$

where $\tau _j$ are the points in the original time intervals discretization. With this we obtain normalized samples $\left[ \tau _k,z(\tau _{k})\right] _i$ which are now comparable between political candidates.

### Alternative model with jumps

The exponential saturation of the mean square log displacement does not uniquely identify the GOU process. Indeed consider a non-diffusive process that jumps between two discrete states $x \in \{ 1, c \}$ with symmetric rate *r*. Its conditional probability $p \equiv p(x(\tau ) = \left. 1 \right||x(0) = 1)$ evolves with $\partial _t p = r\left( 1-2p\right)$ which gives $p=\left( 1+e^{-2r\tau } \right) / 2$ from the initial condition $p=1$. We immediately find $\mathbb {E} \left[ s(\tau )\right] = \frac{\left( \ln c\right) ^2}{2} (1-e^{-2 r \tau })$, which means that the exponential saturation is obtained also by a qualitatively different process not based on Brownian motion, and potentially many more examples are possible. There is therefore a degeneracy in the models that fit the exponential curve of Eq. (7). This degeneracy can be resolved by the distribution of the squared log increments, as for example, this is smooth in the GOU case while it is a single peak in the jump process described above. In this simple jump process and also in more general processes combining diffusion with jumps of uncertain size, the distribution of squared log increments will be characterized by a fat tail for small intervals $\tau$ because jumps are statistically incompatible with continuous diffusion.

### Anomalous diffusion

As mentioned above, the linear scaling of the squared log displacement for small time intervals $\tau$ is consistent with multiple types of diffusion and jump processes. One may ask what model processes instead do not follow this linear scaling law, and the answer is in the theory of anomalous diffusion^47–49^. We argue that this latter more complicated framework is not necessary for our data as we show that, if the twitting rate is filtered with a large enough timescale $\beta ^{-1}$ and for a sizeable range of this parameter, the process is well described by a standard diffusion like the GOU process.

### Checking the distribution

After the parameters $\widetilde{\sigma }_i$ and $\widetilde{\alpha }_i$ are fitted on the empirical average square log-displacement, we can dig a bit deeper and check if the empirical probability distribution of the square log displacements $s_i(\tau _j)$ is comparable with that of the GOU, which is a chi-squared distribution being the square of a Gaussian. For looking at this on a comparable scale between political candidates we consider the normalized increments

10 $$\tilde{z}_{i} (\tau _{j} ) = {\text{ }}\frac{{s_{i} (\tau _{j} )}}{{\left. {{\mathbb{E}}\left[ {s(\tau _{j} )} \right]} \right|_{{\tilde{\sigma }_{i} ,\tilde{\alpha }_{i} }} }}.{\text{ }}$$

### Further detrending

We wish to use as few parameters as possible to keep the description simple, so we do not add additional detrending on the share of attention *x*(*t*) to realize the main text figures. For the sake of completeness, however, we observe that for some political candidates, and especially for the case of the 2022 elections, where our observation period is shorter, a non-negligible drift in popularity is found over the whole period under analysis. This could confound the fit to the mean-reverting process, as the long-range variation is interpreted as a longer correlation time $\alpha ^{-1}$. For this, we introduce a simple detrending of the form $a_i e^{b_i (t-t_0)}$ which improves the fit to the GOU as is shown in the supplementary figures. For our data this period-long drift results to be small enough that the GOU fit to the square log-displacement is good even without detrending when the time intervals considered are between a few minutes and up to a few days. This limit is taken also in order to have enough samples for estimating the parameters $\alpha$ and $\sigma$, and accordingly the calibration intervals $\tau$ should be much shorter compared to the full time span of the data. Furthermore, note that the curve calibration does not involve the average $\widetilde{\mu }$ as seen in Eq. (7). Therefore it seems reasonable when performing predictions to estimate $\widetilde{\mu }$ from an interval of around the calibration timescale rather than the whole history.

## Results

### Logarithmic fluctuations and mean reversion

The share of attention to Italian political parties on Twitter has already been shown to be a good indicator of election outcomes^30,31^. We here discuss how a description in terms of the simple GOU process, which is a model of mean-reverting diffusion in logarithmic scale discussed in the Methods section, fits the short-term empirical fluctuations in the share of attention derived from our data for the 2013 and 2022 Italian political elections.

The fit of the mean square log displacement to the GOU theoretical curve gives the parameters in table 1 for the four main parties in 2013 for which the statistics is sufficient. $\widetilde{\alpha }$ is the mean-reverting parameter and $\widetilde{\sigma }$ is the volatility parameter controlling the amplitude of fluctuations. We see that the timescale of mean-reverting is around $\widetilde{\alpha }^{-1} \approx 3$ hours for all parties, which gives an idea of the typical reaction and debate time on Twitter to incoming news. This mean-reverting timescale estimate of roughly three hours results to be stable under changes in the filtering timescale $\beta ^{-1}$, see supplementary Fig. A1. The volatility results to be larger for the M5S five-star movement party, and this can partly be explained by their twitting rate being smaller and absolute variations being amplified in logarithmic scale.

**Table 1:** Parameter fit for the GOU process in the 2013 italian political elections, with a filtering timescale of $\beta ^{-1} = 20$ min.

Political party	$\widetilde{\alpha }$	$\widetilde{\sigma }$
PD	0.301	0.282
SC	0.298	0.235
PdL	0.255	0.239
M5S	0.297	0.368

**Figure 1:**
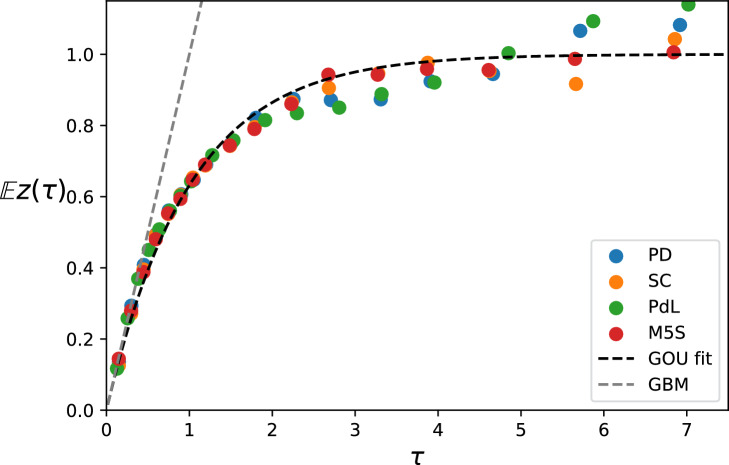
Fit to the GOU process Empirical mean square log-displacement in normalized units for the four main political parties in the Italian 2013 elections, and the corresponding theoretical curves. The filtering timescale used for the instantaneous twitting rate is $\beta ^{-1} = 20$ min. We see that the GOU model, a mean-reverting diffusion process in logarithmic scale with just two parameters is able to fit the empirical curves on short timescales. GBM is Geometric Brownian motion. Precise definitions in the Methods section.

By normalizing the twitting rate and rescaling time according to the obtained GOU fit, we visualize the structure of the fit in Fig 1. Some slight deviation from the theoretical curve suggests the presence of nonlinearities or some drift structure visible already at the calibration timescale. Actually, it could seem like a rather strong assumption for the twitting rate to fluctuate around an underlying average which is constant during the few weeks preceding the elections, as a political campaign typically intensifies when approaching election dates exactly to shift the political attention towards their candidates. Indeed our calibration is limited to timescales between a few minutes up to a few days so that trends on longer timescales are not considered. For our particular dataset, however, we find that removing the trend with a simple exponential fit does not substantially improve the GOU curve fit of Fig. 1, see Supplementary Fig. A2, meaning that the drift structure is more complex than our single-timescale description. Further characterizing these effects is not statistically feasible with our small dataset, and we consider the minimal description in terms of the GOU process appropriate and the fit in Fig. 1 satisfactory.

**Figure 2:**
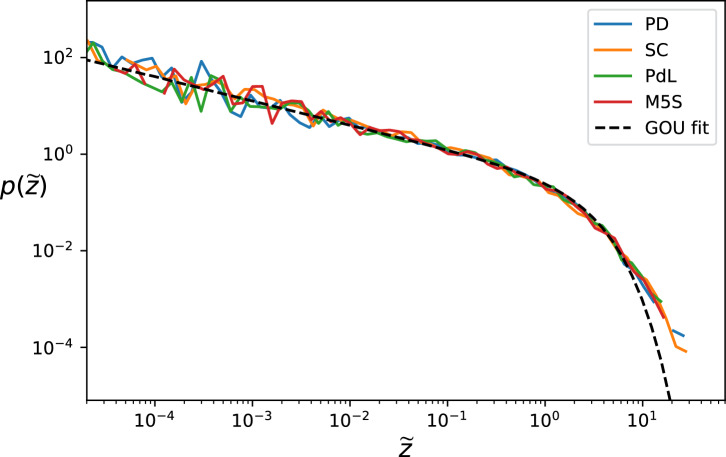
Comparing the distribution. Empirical distribution of the squared log-displacement in normalized units and comparison to the GOU model fit from Fig. 1. The fat tails are explained by few extreme events. Python “powerlaw”^50^ package was used for plotting. The time interval is $\tau =180$ min, larger than the filtering timescale and comparable with the mean-reversion timescale.

Regarding the distribution of the squared log-displacement, the GOU process would prescribe it to be a chi-squared distribution. In Fig. 2 we see some deviation from the theoretical curve at the large values tail, which cannot be explained from the diffusive statistics and are more properly described as jumps. Indeed, as discussed in the Methods section, also a jump process can have a mean square log-displacement analogous to the GOU process, and the degeneracy is exactly removed by looking at the distribution. However, these jumps happen in a relatively small number and it is not feasible to add more parameters to model a jump distribution on top of the diffusion from this small dataset, and we therefore consider these just as outliers or extreme events.

Let us also mention that we observe some skewness in the log-displacement distribution which is understood as typically the reaction to events is faster than the relaxation from the excited states.

### Extreme events

Let us here consider some of these large twitting rate jump events, as they reflect the community’s reaction to the most impactful news.

The first one we list happened on the 12/02/2013 and affected the PdL party of Silvio Berlusconi. In particular, a satirical comedian well-known in Italy went to the Sanremo music festival for a comedy segment which particularly targeted Berlusconi. Some in the audience started shouting and interrupted the comedian, which sparked a broader discussion in the public opinion ad likely also on Twitter about free speech and the appropriateness of political satire in the national music festival.

Another one regards the PD democratic party when on the 07/01/2013 the candidate president Pierluigi Bersani was hosted in the popular television program “Otto e Mezzo” to discuss his political agenda. The following day Berlusconi participated in the same tv program and a comparable spike happened in the share of attention.

From the definition we see that the events which impact most the square log-displacement on the shortest timescale $\beta ^{-1}$ are the unexpected news. As an example consider the very popular tv debate between Berlusconi and its most critical journalist Marco Travaglio, and while it provoked a massive debate and interest on Twitter, the program was relatively long and it was planned weeks ahead, so that the Twitter reactions spread over a longer period of time so that twitting rate variations were relatively moderate.

Another event we discuss regards the SC party, that from its official Twitter account in the morning of 15/02/2013 revealed that their candidate Mario Monti had been secretly offered important political roles from other parties in exchange for him renouncing to run for president. Understandably, this sparked quite an immediate reaction.

**Figure 3:**
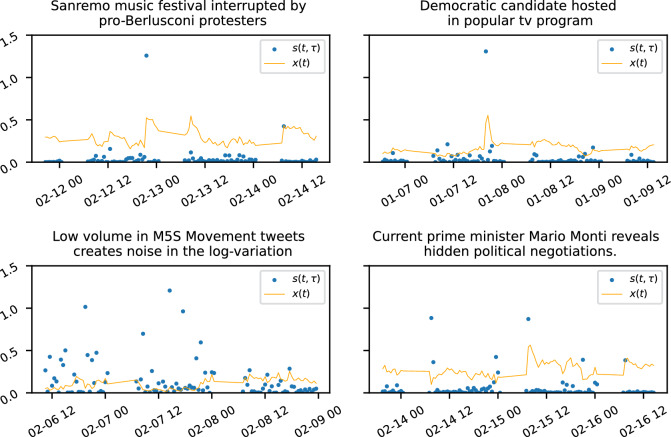
Examples of extreme events. *x*(*t*) is the share of attention at time *t*, and $s(t,\tau )$ is the corresponding squared log-displacement, here for an interval $\tau = 30$ min and with filtering timescale $\beta ^{-1}= 20$ min. We see how significant and unexpected events in the electoral campaign are reflected in spikes of *x*(*t*).

We note that for the anti-establishment five star movement, which actually was the surprise of the elections results, Twitter was not the main platform for propaganda and discussion as they did use the movement’s website. As a consequence, the twitting rate was often so low that small absolute variations can have a big impact in the logarithmic variations, as is shown in Fig. 3 together with the other events discussed here.

### What changed in 2022

We have a much shorter dataset for the 2022 elections, and only for the two weeks preceding the elections which were strongly characterized by two events, namely the television debate between the two main candidates Enrico Letta and Giorgia Meloni, and the strong personal accusations between the other two candidates Matteo Renzi and Giuseppe Conte. These two events concentrated much of the public attention in our data timeframe, and as a consequence we observe a lack of saturation and the fitted timescale of mean-reverting is larger (around two times) compared to the 2013 elections. The fit to the GOU process is still satisfactory but less accurate, see Supplementary Fig. A3.

### Out-of-sample predictions

**Figure 4:**
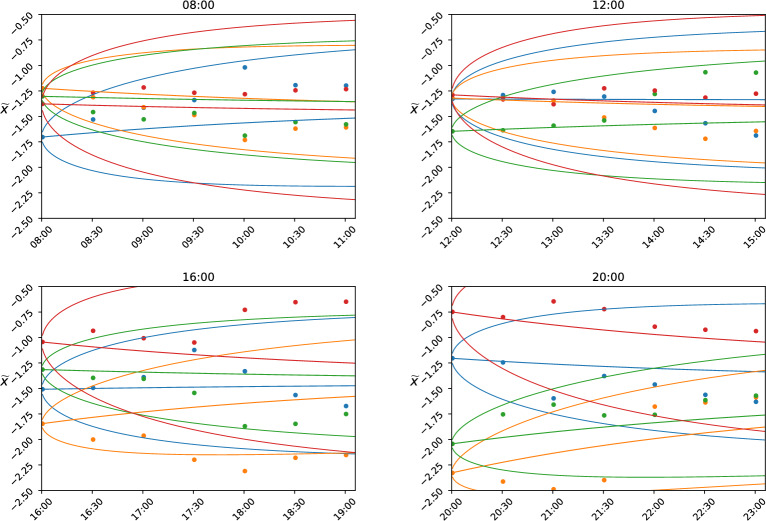
Predictions using the GOU model. Time series predictions based on the GOU model with the parameters estimated immediately before prediction, at four different times during the day of 22 February 2013. The scatter plot is the realized $\widetilde{x}$, while the three solid lines are the expectation and ± 2 standard deviations. The colors are as above: blue is ’PD’, orange is ’SC’, green is ’PdL’, red is ’M5S’.

We study the dynamics of attention on short timescales and in this limit we identify fluctuations and mean-reversion as the two main dynamical features. The GOU model can then be used for predicting the attention dynamics on timescales comparable to the mean-reverting timescale $\alpha$, which was here estimated to be of around 3 h. As an example, we consider the last day before the elections 22 February 2013 (23 Feb was pre-election silence) at four different time instants, and visualize the expectation and standard deviation obtained from the GOU model. The parameters are obtained as above but only from data up to the prediction time instant, and with an instantaneous log-mean $\widetilde{\mu }$ for mean-reversion estimated from the previous 40 hours which is the largest calibration interval. The results are shown in Fig. 4. The well-known expectations of the Ornstein–Uhlenbeck process^46^ are such that, given an initial state $\widetilde{x}(0)$, the expected state after a time interval $\tau$ is $\mathbb {E} \left[ \widetilde{x}(\tau ) | \widetilde{x}(0)\right] = \left( \widetilde{x}(0) -\widetilde{\mu } \right) e^{-\alpha \tau } + \widetilde{\mu }$, and its variance is $\textrm{Var} \left[ \widetilde{x}(\tau ) | \widetilde{x}(0)\right] = \frac{\sigma ^2}{2\alpha } (1-e^{-2\alpha \tau })$.

## Discussion

In this paper we present an analysis on two distinct dataset of Twitter messages time series corresponding to different Italian political elections. Our study reveals that, on short timescales from a few minutes to a few days, the dynamics of political discussions on Twitter exhibit characteristics consistent with a Geometric Ornstein–Uhlenbeck (GOU) evolution, that is a linear mean-reverting diffusion process on a logarithmic scale. Crucially, this minimal description involves a single timescale which we estimate from our data to be of around three hours. Small discrepancies from the fitted curve reflect the non-modeled multiscale properties of the dynamics. This unique modeling choice enables us to capture the nuanced patterns of attention and opinion exchange in the ever-changing landscape of political conversations. The approach presented here contributes to the problem of modeling short-term fluctuations in Twitter attention by estimating a timescale of mean-reversion which enables the detection of changes in the location of the longer-term process net of short-lived fluctuations; how to model election outcomes as a function of this Twitter attention relies instead on other studies^35,51^. We have here been dealing with a politics-only dataset, therefore we cannot establish whether the GOU model is peculiar of the political attention or rather a more general form of attention dynamics.

One notable aspect of our analysis is the identification of extreme events within the political Twitter data. These events, characterized by significant deviations from the linear mean-reverting behavior, underscore the non-linear and unpredictable nature of political discussions on the platform. Understanding and quantifying these extreme events are crucial for a comprehensive analysis of the evolving political discourse. The application of our findings in election forecasting holds substantial promise. By incorporating the GOU evolution model, we gain a more nuanced understanding of how political sentiments and attention levels fluctuate over time. This insight can enhance the accuracy of election predictions by accounting for the inherent volatility in public opinion, especially during critical periods such as debates, policy announcements, or unexpected events. Indeed, the GOU model allows for real-time monitoring of Twitter data, providing a dynamic and adaptive framework for assessing the ebb and flow of political discussions. This real-time capability enables us to identify emerging trends and potential turning points in public opinion, offering valuable insights for election forecasting models that seek to capture the zeitgeist of the electorate. Our approach complements the studies of the attention to politicians on longer timescales where fluctuations become negligible and the dynamics is described by trends and inertial effects^31,35–41^. While our study presents a novel framework for understanding political Twitter dynamics, there are challenges and avenues for further exploration. Future research could delve into refining the GOU model parameters and especially determining if the three hours timescale identified here changes in other datasets and situations, and more in general investigating the transferability of the model across different political landscapes.

## Supplementary Information

Supplementary Information 1.

Supplementary Information 1.

Supplementary Information 1.

Supplementary Information 2.

Supplementary Information 3.

## Data Availability

The 2013 Data are available from Figshare as published in Ref.^31^, http://dx.doi.org/10.6084/m9.figshare.1437740. The 2022 dataset is available from the corresponding author on reasonable request. The python code developed for the data analysis in this manuscript is publicly available on https://github.com/AndreaAuconi/Twitter_time_series_analysis.
