# Supplementary material for: Fluctuations and extreme events in the public attention on Italian legislative elections
Source: Sci Rep. 2024 Oct 1;14:22804. doi: 10.1038/s41598-024-69354-y (PMC11445506; doi:10.1038/s41598-024-69354-y)
Supplement: Supplementary file 2 — Supplementary Information 1. [file 41598_2024_69354_MOESM2_ESM.docx]

**Keywords 2013**

**PD:** pd, pdnetwork, pbersani, bersani

**SC:** scelta_civica, SenatoreMonti, monti

**PdL:** forza_italia, berlusconi, forza italia

**M5S:** Mov5Stelle, luigidimaio, di maio, grillo

**Keywords 2022**

**Calenda:** carlocalenda, calenda, azione

**Letta:** enricoletta, letta, pdnetwork

**Meloni:** giorgiameloni, meloni, fratelliditalia

**Conte:** giuseppeconteit, conte, stelle

**Renzi:** matteorenzi, renzi, italiaviva

**Salvini:** matteosalvinimi, salvini, lega, legasalvini
