# Supplementary material for: Fluctuations and extreme events in the public attention on Italian legislative elections
Source: Sci Rep. 2024 Oct 1;14:22804. doi: 10.1038/s41598-024-69354-y (PMC11445506; doi:10.1038/s41598-024-69354-y)
Supplement: Supplementary file 4 — Supplementary Information 2. [file 41598_2024_69354_MOESM4_ESM.pdf]

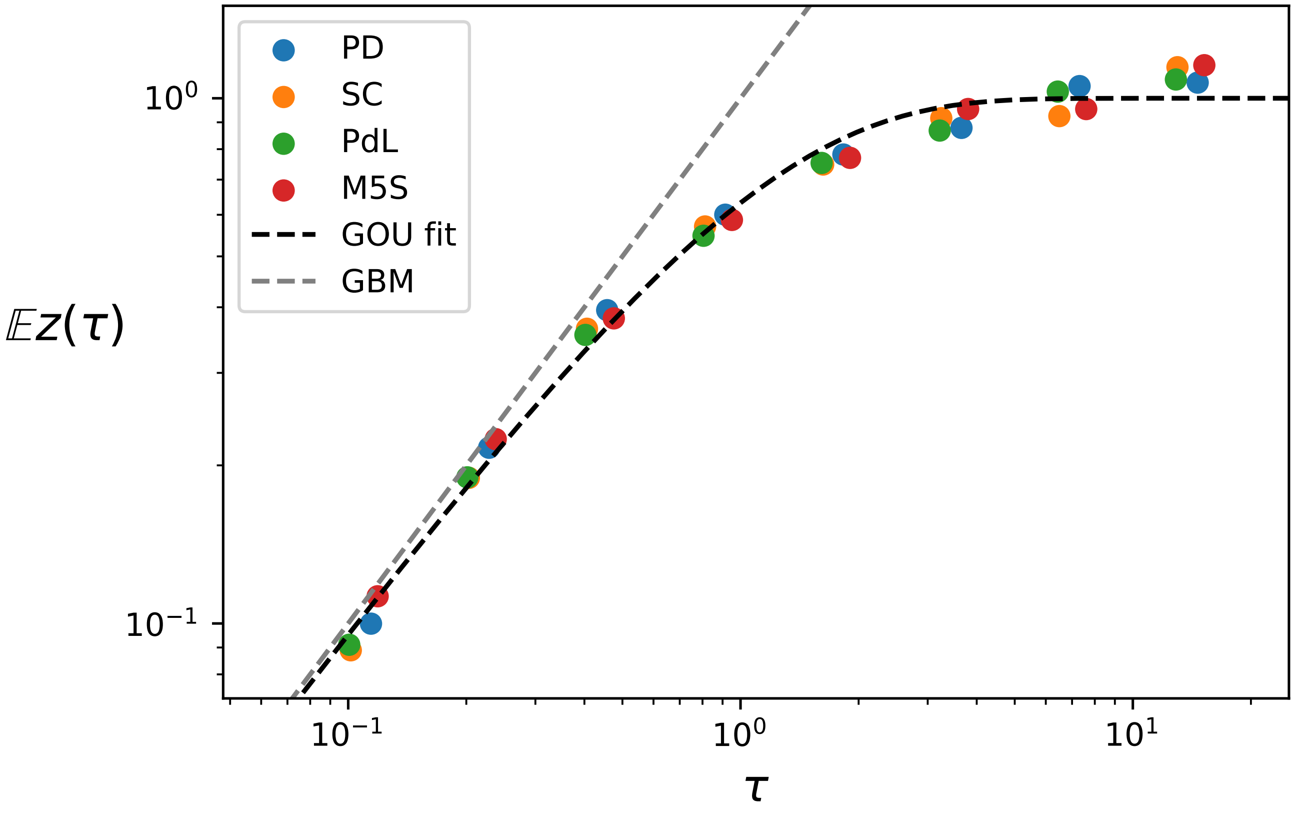

Supplementary Figure A2: Fit to the GOU process. Empirical mean square log-displacement in normalized units for the six main political leaders in the Italian 2013 elections after detrending, and the corresponding theoretical curves. The discretization time used for the instantaneous twitting rate is $\beta^{-1} = 20min$. GBM is Geometric Brownian motion. Precise definitions in the Methods section.
