# Supplementary material for: Fluctuations and extreme events in the public attention on Italian legislative elections
Source: Sci Rep. 2024 Oct 1;14:22804. doi: 10.1038/s41598-024-69354-y (PMC11445506; doi:10.1038/s41598-024-69354-y)
Supplement: Supplementary file 5 — Supplementary Information 3. [file 41598_2024_69354_MOESM5_ESM.pdf]

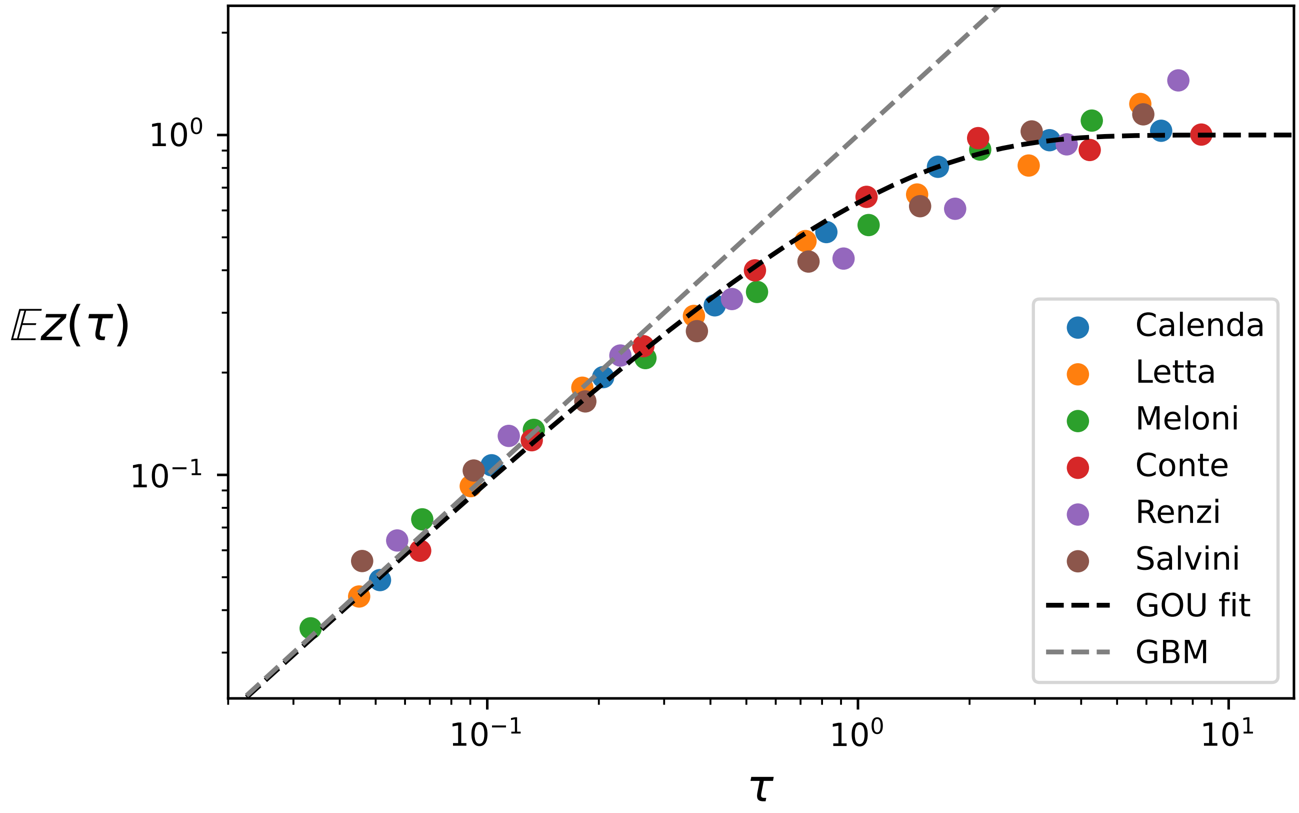

Supplementary Figure A3: Fit to the GOU process. Empirical mean square log-displacement in normalized units for the six main political leaders in the Italian 2022 elections, and the corresponding theoretical curves. The discretization time used for the instantaneous twitting rate is $\beta^{-1} = 20min$. GBM is Geometric Brownian motion. Precise definitions in the Methods section.
